# Infra-Low Frequency Neurofeedback rapidly ameliorates schizophrenia symptoms: A case report of the first session

**DOI:** 10.3389/fnhum.2022.923695

**Published:** 2022-09-21

**Authors:** Joannis N. Nestoros, Nionia G. Vallianatou

**Affiliations:** Electrophysiological Prometheus Laboratories, Synchronal Amphiaraia, University of Crete, Athens, Greece

**Keywords:** schizophrenia, Neurofeedback, antipsychotic treatment, Infra-Low Frequency Regime, rapid improvement

## Abstract

A 38-year-old army officer started therapy in 2020 with a four-year history of auditory hallucinations and delusions of reference, persecution and grandeur, symptoms that were resistant to traditional antipsychotic medications. He follows an integrative psychotherapy program that aims to reduce his anxiety, continues his antipsychotic medications, and has Infra-Low Frequency Neurofeedback. After his initial assessment he had a 40 min session of Infra-Low Frequency Neurofeedback before any other kind of intervention. Before and immediately after the session he completed the SCL-90 scale and the Visual Analog Scale covering 20 aspects of his psychological and physical state as well as his schizophrenic symptoms. This first Neurofeedback session had dramatic effects on his psychotic symptoms, levels of anxiety and psychosomatic condition, before his first psychotherapy session and/or any changes in his antipsychotic medication. The above results have great importance due to the severity and chronicity of schizophrenia. Informed consent was obtained from the participant for the publication of this case report (including all data and images).

## Introduction

Schizophrenia is without doubt one of the most fascinating topics of research because it involves biological, psychological, family and sociocultural factors. The evolution of the concept of schizophrenia since 1860 and its effects on current thinking has been discussed by Nestoros (1997a,b) as well as by others (Tandon et al., 2010; Keshavan et al., 2011). In summary, whereas we previously had in the scientific literature at least 26 different types of schizophrenia (Nestoros, 1997a,b, 2012a,b), we ended in DSM-5–first published in 2013–with only one type of schizophrenia characterized as a severe, chronic mental disorder with disturbances in thought, perception and behavior that resembles what was previously called schizophrenia, paranoid type.

In our opinion, certain two neurophysiological models for the etiology of schizophrenic symptoms that relate to one classical neurotransmitter, which is γ-aminobutyric acid (GABA) (Roberts, 1972, 1976), and one neuropeptide/neuromodulator which is cholecystokinin-CCK (Hockfelt et al., 1980a,b), although about 40 years old, when combined with very recent relevant research (Whissell et al., 2015, 2019; Ballaz and Bourin, 2021a,b; Ballaz et al., 2021; Ochoa-de la Paz et al., 2021), prove their contribution to understanding schizophrenia. The two models are not in conflict but complement each other Nestoros, 1980a. Firstly, the increase of extreme stress causes the hyperactivity of dopaminergic (Brisch et al., 2014) and the hypoactivity in GABAergic neurotransmission (Nuss, 2015). Diazepam is known to reduce dopamine release in nucleus accumbens (Gomez et al., 2017) and potentiates the inhibitory action of GABA (Haefely, 1978; Haefely et al., 1978a,b; Costa and Guidotti, 1979; Nestoros and Nistri, 1979; Nestoros, 1980b,c, 1982, 1997a,b); while cholecystokinin regulates dopaminergic (as well as GABAergic and glutaminergic) activity through a corticolimbic and a mesolimbic dimmer-switch process (Ballaz and Bourin, 2021a,b; Ballaz et al., 2021). The inhibitory action of γ-aminobutyric acid (GABA) is mediated predominantly by interneurons (Kelsom and Lu, 2013). The interneurons have a crucial role in neural processing, impinging on behaviors such as fear, social interaction, anxiety, locomotion, cognition, information processing and memory. So, dysfunctions in interneuron signaling affect the behavioral functions and have implications to the many psychiatric and neurological disorders (Ochoa-de la Paz et al., 2021). Interneurons have many subtypes and contribute differentially to behavioral patterns in health and disease. One classification of interneurons is perisomatic interneurons that can be divided into two different groups based on the expression of the molecular markers cholecystokinin (CCK-GABA neurons) and parvalbumin (PV-GABA neurons) (Whissell et al., 2015). CCK-GABA neurons are considered to be involved in the regulation of mood, anxiety and fear, since their function has been largely attributed to a modest population of neurons in amygdala (Vereczki et al., 2021). CCK-GABA neuron distribution is also localized in several regions of the hippocampus, thalamus and cortex (Whissell et al., 2019).

## Case report

P. H. is a male, right-handed, 38-year-old unmarried army officer. He had a four-year history of auditory hallucinations and delusions of reference, persecution and grandeur, symptoms that were resistant to traditional antipsychotic medications (Clozapine 700 mg per day, Amisulpride 600 mg per day, Valproic acid 1,000 mg per day and Fluoxetine 40 mg per day). Even though the medications did not resolve the issues, this regime was maintained throughout, and continued unchanged through the neurofeedback training. The patient complied with DSM-5 and ICD-11 (2022) criteria for schizophrenia when assessed by the Army psychiatrists and by our center. He experienced the first auditory hallucinations during his adolescence. He started therapy in December 2020. In his initial evaluation he complained of the consistent presence of “loud voices” and many stereotypic behavior patterns that he was forced to execute because of these voices, such as whispering psalms, and/or quick glance movements. He was started on integrative psychotherapy, which aims to reduce his anxiety, continued his antipsychotic medications, and was started an Infra-Low Frequency Regime Neurofeedback following the Othmer and Othmer (2016, 2017, 2020) methodology.

After his initial assessment and before any other kind of intervention, he had a 1 h session of Infra-Low Frequency Neurofeedback. Before and immediately after the session he completed the SCL-90 scale (Derogatis, 1977) and the Visual Analog Scale, covering 20 aspects of his psychological and physical state as well as his schizophrenic symptoms (Nestoros, 1998). He was instructed to complete the scales answering to the time period “How I feel now”? This first Neurofeedback session had dramatic effects on his psychotic symptoms, levels of anxiety and psychosomatic condition, before his first psychotherapy session and/or any changes in his antipsychotic medication. This positive result convinced him to continue the psychotherapeutic and neurofeedback sessions until today. He presently reports (end of March 2022), after eighteen (18) neurofeedback sessions, minor levels of transient and manageable auditory hallucinations and delusions only under stress conditions, without stereotypic behavior patterns. He enjoys his administrative duties, assigned by the army, and 6 months ago started to date a young woman, moving away from his mother's home, where he had been living before. His antipsychotic medication remained the same throughout the above course of treatment, since the army psychiatrists felt that he was doing so well that there was no reason to change it.

## Methodology

**Figure 1:**
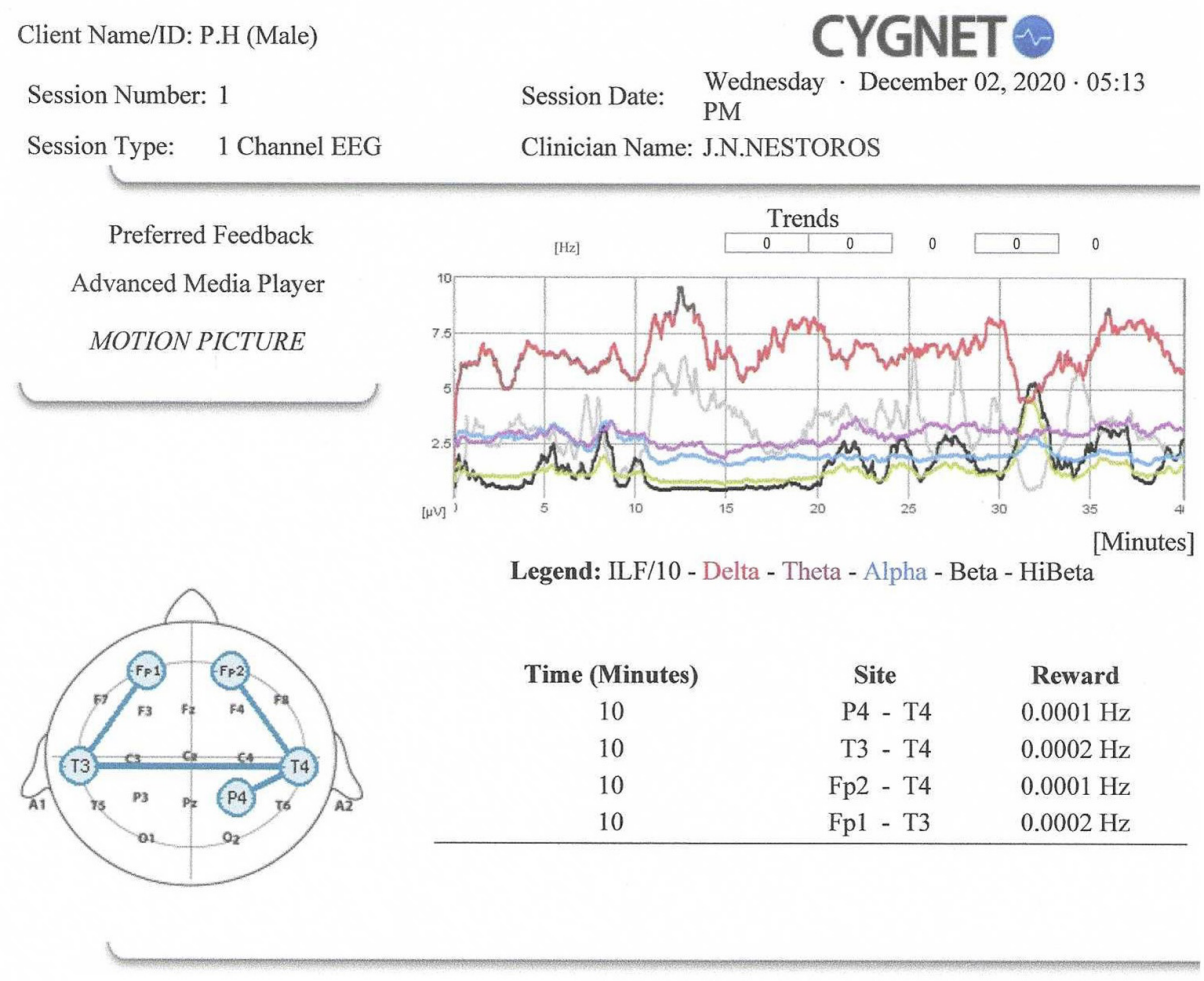
Neurofeedback session report.

Informed consent was obtained from the participant for the publication of this case report (including all data and images). We have placed the electrodes (recording, reference and ground electrodes) in particular areas of the head in accordance with the “Ten percent electrode system for recording EEG activity” (Chatrian et al., 1985) which is based on the “Ten-twenty electrode system” which was proposed by Jasper (1958). The placement of the electrodes and the time of Infra-Low Frequency Neurofeedback employed is shown on Figure 1. We trained P4-T4 (0–10 min), T3-T4 (10th−20th min), Fp2-T4 (20th−30th min and Fp1-T3 (30th−40th min for a total of 10 min each, in the above order. For the right hemisphere reward frequencies were set to 0.0001 Hz and for the left hemisphere at 0.0002 Hz, with percent success of multiple inhibits (with 10 inhibit bands covering the spectrum to 40 Hz) maintained at 95%. That is, the level of difficulty was kept at a level such that inhibits were engaged about 5% of the time. The Othmer and Othmer (2016, 2017, 2020) neurofeedback methodology was followed, using Cygnet Software and Bee Medic one-channel EEG Advance Media player. The patient watched a motion picture while his ILF/10, Delta, Theta, Alpha, Beta and HiBeta band activity was continuously monitored. The patient was attentive throughout the entire session. Before and immediately after the session he completed the SCL-90 (Derogatis, 1977) and the Visual Analog Scale (Nestoros, 1998) covering 20 aspects of his psychological and physical state as well as his schizophrenic symptoms. He was asked to complete both above Scales according to how he was feeling “now”.

## Results

As shown on Figure 1, Delta band activity predominated and was higher in all recorded areas throughout the session. ILF/10 amplitude ranked 2nd and was significantly lower than Delta. came Theta band activity, ranked 3rd, followed by Alpha. HiBeta amplitude was the lowest, with one exception between the 30th and the 33th min, at the beginning of the period of recording Fp1-T3, possibly because of anxiety-producing thoughts that were reflected in left frontal lobe activity.

**Figure 2:**
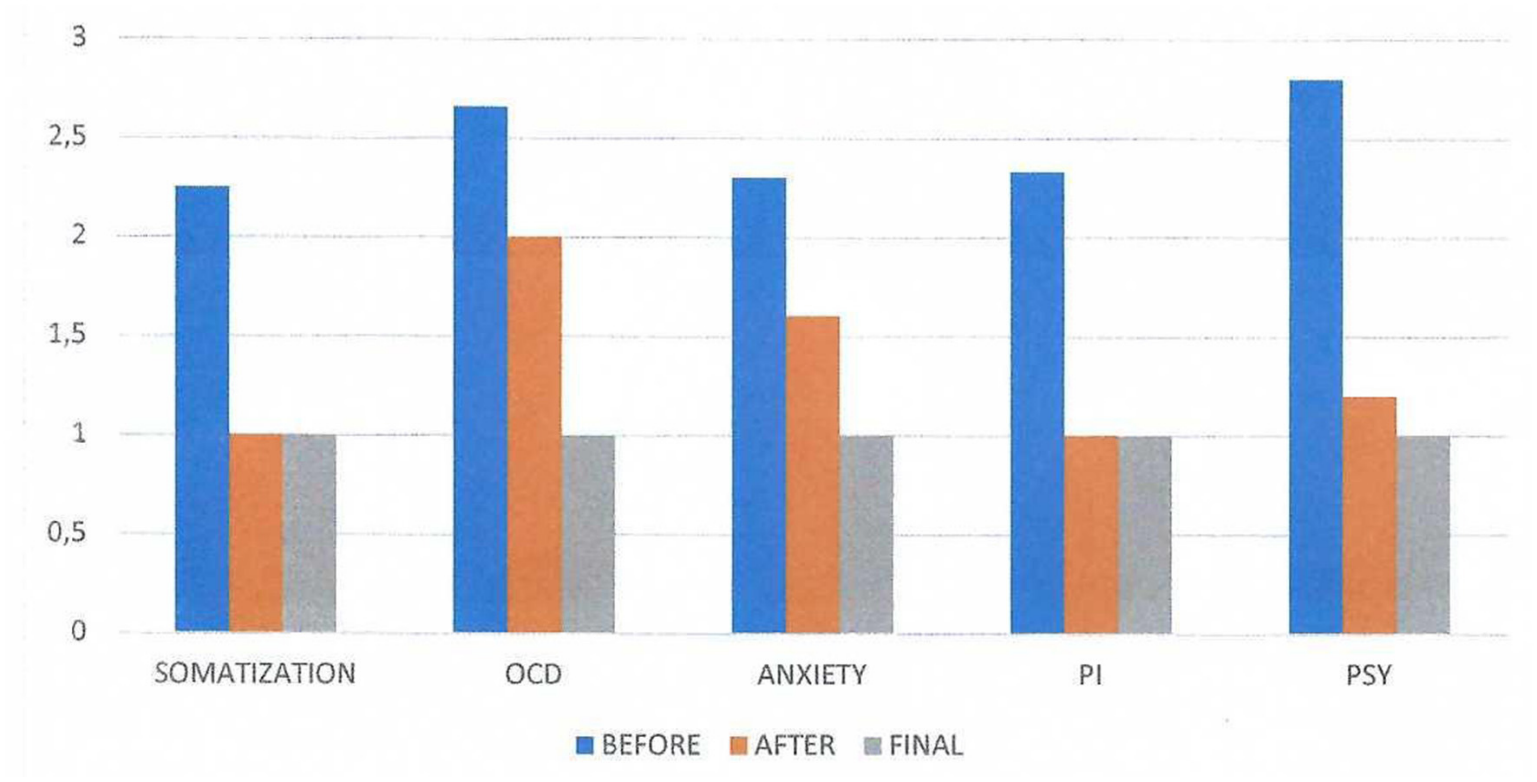
SCL-90 results on three conditions: before and after the first neurofeedback session and the results of the final session. The term Somatization represents the somatic symptoms that were reported by the subject before the session. There was no difference in the appearance of these symptoms after the session. The abbreviation OCD represents the obsessive-compulsive symptoms before and after the session, the abbreviation PI represents the paranoid ideation before and after the session and the abbreviation PSY represents the Psychoticism (that is the psychotic symptoms) before and after the session. We evaluated the mean scores of each factor and we can observe significant variation in the factors “Anxiety”, “Paranoid Ideation” and “Psychoticis”.

Figure 2 shows the SCL-90 results before and after the first neurofeedback session. The term Somatization represents the somatic symptoms that were reported by the subject (headache, dizziness, pain in the heart or chest, nausea or upset stomach, etc.). The abbreviation OCD represents the obsessive-compulsive symptoms (“Unwanted thoughts, words or ideas that cannot leave your mind”, “Worrying about sloppiness or carelessness”, “Having to check and recheck what you do,” etc.); the abbreviation PI represents the paranoid ideation (“Feeling that others are to blame for most of your problems”, “Feeling that most people are not trustworthy”, “Feeling that other people watch you or talk about you”); and the abbreviation PSY represents the Psychoticism (that is the psychotic symptoms such as “The idea that somebody controls my thoughts”, “Hearing voices that other people do not hear”, “Other people can read my thoughts,” etc.). We evaluated the mean scores of each factor, and we can observe significant post-session differences in the factors “Anxiety”, “Paranoid Ideation” and “Psychoticism”.

**Figure 3:**
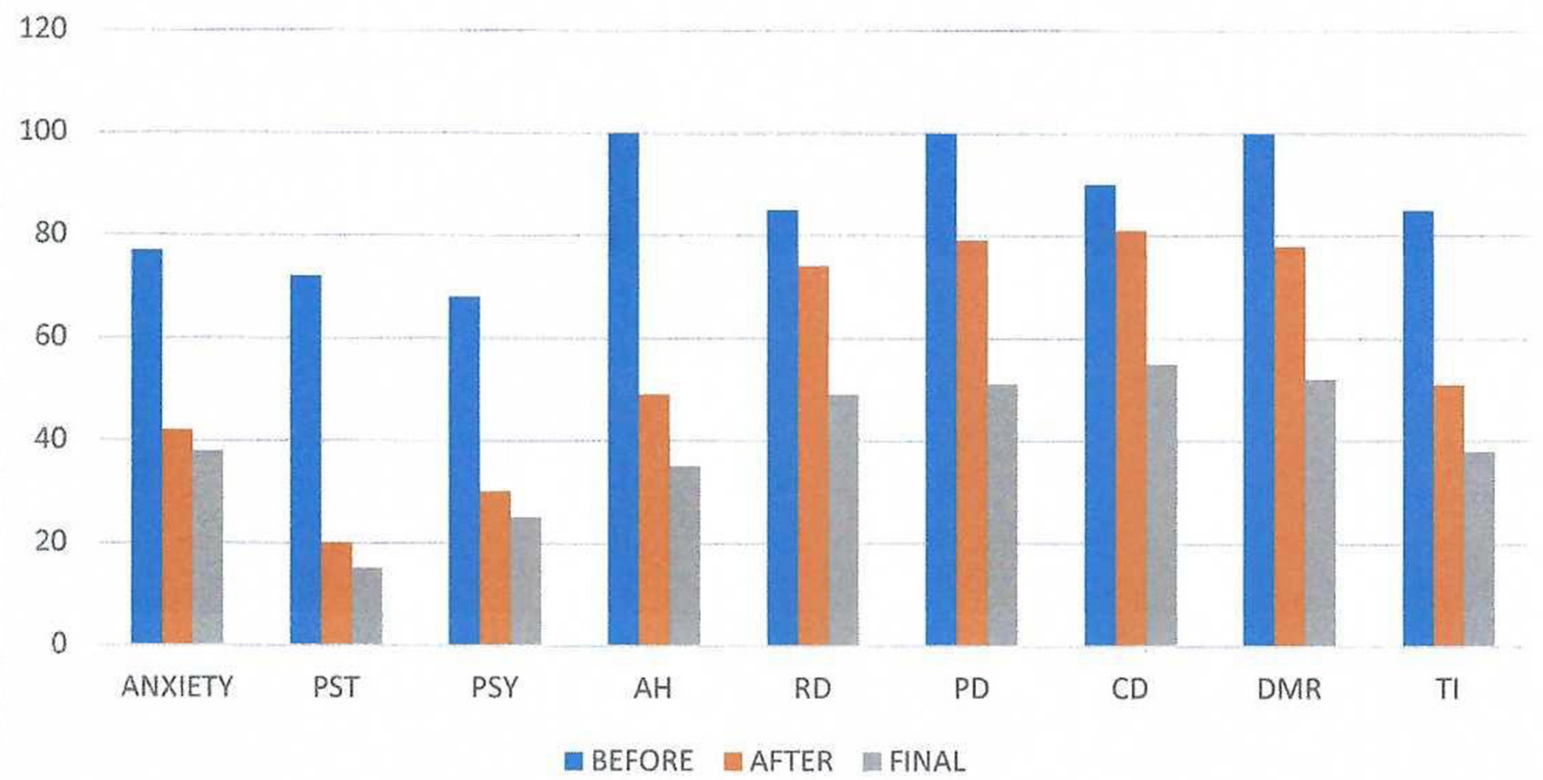
Visual Analog Results on three conditions: before and after the first neurofeedback session and the results of the final session. The abbreviation PST represents the general psychological state of the subject, PSY the psychosomatic symptoms, AH the auditory hallucinations, RD the delusions of reference, PD the persecutory delusions, CD the delusions of being controlled by outside forces, DMR the delusions of mind reading, and TI the thought insertion. The self-assessment scale had a length of 10 cm, where “O” represented “I have no such symptom” and “I00” represented “The symptom is extremely annoying”. We evaluated the mean scores. We observe great significance in the scales AH, PST, Anxiety, DMR, and TL.

Figure 3 shows the results obtained by using the Visual Analog Scale (Nestoros, 1998) covering 20 aspects of his psychological and physical state as well as his psychotic symptoms (negative and positive) according to Andreasen (1984a,b). The abbreviation PST represents the general psychological state of the subject, PSY the psychosomatic symptoms, AH the auditory hallucinations, RD the delusions of reference, PD the persecutory delusions, CD the delusions of being controlled, DMR the delusions of mind reading, and TI the thought insertion. The self-assessment visual analog scale had for every item a vertical line 10 cm in length, where “0” represented “I have no such symptom” and “100” represented “The symptom is extremely annoying”. We evaluated the mean scores before and after the first neurofeedback session. We observe significant positive changes in the scales AH, PST, Anxiety, DMR and TI.

## Discussion

According to a review of recent meta-analyses, the acute efficacy in schizophrenia was found to be modest (Haddad and Correll, 2018) with the antipsychotic drugs that were developed as dopamine D2 postsynaptic receptor antagonists (Seeman and Lapur, 2000). Furthermore, according to Heinz E. Lehmann, the McGill professor who pioneered the use of antipsychotic drugs in North America (Stip, 2015), schizophrenic delusions were affected between the fourth and sixth week of treatment. It was therefore an extremely pleasant surprise to observe schizophrenic patients improve to the point of being symptom-free within hours or a few days of high doses diazepam treatment (Nestoros et al., 1982). The results were so impressive that all patients were videotaped at baseline before treatment and within the next 24 h, in order to provide material for other colleagues to verify our findings. These results when presented at the Annual Meeting of the American Psychiatric Association, 2013, Toronto 1982, made headlines on Medical and other newspapers all over the world. Consequently, we found that high doses of diazepam improve neuroleptic-resistant chronic schizophrenic patients (Nestoros et al., 1983). In parallel with the benzodiazepines in schizophrenia studies, our research group studied the effects of CCK-33 in schizophrenic patients (Nair et al., 1982) and CCK-8 in neuroleptic-resistant schizophrenic subjects (Nair et al., 1983, 1985). Again, we documented on videotapes severe schizophrenic symptoms to improve 1 h after CCK intravenous administration.

The optimism resulting from the above psychopharmacological studies led the first author of the present paper to propose a novel Integrative Psychotherapy model for schizophrenia and other psychoses (Nestoros, 1997a,b, 2001, 2006, 2018) which was rigorously tested (Kalaitzaki and Nestoros, 2006; Zgantzouri et al., 2006; Kalaitzaki et al., 2009, 2010; Nestoros et al., 2016; Zgantzouri and Nestoros, 2017). Moreover, the first author (J.N.N.) of this paper has many times treated with success patients suffering from severe schizophrenic symptoms with an integrative approach utilizing psychotherapy, antipsychotic drugs, diazepam and CCK-8 and published these results in Greek in textbooks used by the University of Crete and other Greek Universities (Nestoros, 1993, 2012b). Since 2009, Neurofeedback was added to the integrative treatment of patients exhibiting schizophrenic symptoms. The results of the present case study of the first Neurofeedback session signifies its distinct contribution to their improvement.

Obviously, psychotherapy alone needs several months to several years to decrease schizophrenic symptoms and traditional antipsychotics at least several weeks. It is therefore suggested that the beneficial effects of Neurofeedback on schizophrenic symptoms are mediated by GABA and CCK.

Furthermore, a neurophysiological model of anxiety has been proposed (Nestoros, 1980b, 1981, 1984) conceptualizing anxiety as a state of diminished GABAergic neurotransmission resulting from too frequent recruitment of GABAergic neurons. Repetitive stimulation of the recurrent inhibitory pathway in rat hippocampus leads to a remarkable reduction of the effectiveness of GABA, with GABA receptors becoming insensitive to endogenous and iontophoretically applied GABA. This “disinhibition” is probably related to “fading” of the GABA response (Krnjevic, 1980a,b). The above phenomenon is counteracted by antianxiety drugs, including ethanol (Nestoros, 1980b,d). Since in the model of GABA receptor suggested by Costa and Guidotti (1979) an endogenous peptide inhibitor of GABA receptors was included, in view of recent evidence presented here, one can hypothesize that this peptide is CCK. One may speculate with Krešimir Krnjevic (see Ben-Ari et al., 2022) that GABA needs a mechanism to stop it from shutting down the entire nervous system.

The answer as to why neurofeedback benefits such a great variety of different brain dysfunctions may be found in mechanisms similar to the one suggesting that cholecystokinin regulates dopaminergic (as well as GABAergic and glutaminergic) activity through a corticolimbic and a mesolimbic dimmer-switch process (Ballaz and Bourin, 2021a,b; Ballaz et al., 2021).

As for the Delta band activity that was found in the present study to be elicited by the Infra-Low Frequency Neurofeedback, the following thoughts may be of value. Firstly, it is known that Delta waves arise either in the thalamus or in the cortex (Kropotov, 2009). The particular waves can appear during sleep and in wakefulness (Assenza et al., 2015; Frohlich et al., 2021). As Watson (2018) points out in his review of Cognitive and Physiologic Impacts of the Infraslow Oscillation (ISO) (from 0.01 to 0.1 Hz), this rhythm coincides with the oscillation underlying resting state networks (RSNs) in awake human subjects, which are spontaneous fluctuations in brain activity observed with functional magnetic resonance imaging (Fox and Raichle, 2007) and relates the phenomenon of ISOs to the proposal by Halász et al. (2004) that the brain needs to rest at times. Watson (2018) concludes his extensive review of the literature stating: “The ISO typically peaks in power at 0.02 Hz, is seen in multiple brain regions in mammalian species from mice to humans and occurs across wake and sleep. It organizes neurons, oscillatory patterns, sleep patterns, brainwide networks, is modulated by and modulates both pathological conditions and cognitive performance. Furthermore, the thalamus and in particular a glial effect on the thalamus is a candidate for the generation of these rhythms”. The possible involvement of glial cells is supported by the findings of MacVicar et al. (1989) that GABA- activated Cl^−^ channels in astrocytes (a form of glial cells) in hippocampal slices, and by the findings of Deemyad et al. (2018) that astrocytes, although they do not fire action potentials themselves, integrate and drive action potential firing in inhibitory interneuron subnetworks. This indirectly induced firing of action potentials, mediated through GABA-activated Cl^−^ channels in astrocytes, may explain why Infra-Low Frequency Neurofeedback can elicit Delta band activity. This merely implies that the frequency-based organization of resting states extends into the Delta band.

## Data availability statement

The raw data supporting the conclusions of this article will be made available by the authors, without undue reservation.

## Ethics statement

Written informed consent was obtained from the patient for the publication of any potentially identifiable images or data contained within the article.

## Author contributions

All authors listed have made a substantial, direct, and intellectual contribution to the work and approved it for publication.

## Conflict of interest

The authors declare that the research was conducted in the absence of any commercial or financial relationships that could be construed as a potential conflict of interest.

## Publisher's note

All claims expressed in this article are solely those of the authors and do not necessarily represent those of their affiliated organizations, or those of the publisher, the editors and the reviewers. Any product that may be evaluated in this article, or claim that may be made by its manufacturer, is not guaranteed or endorsed by the publisher.
